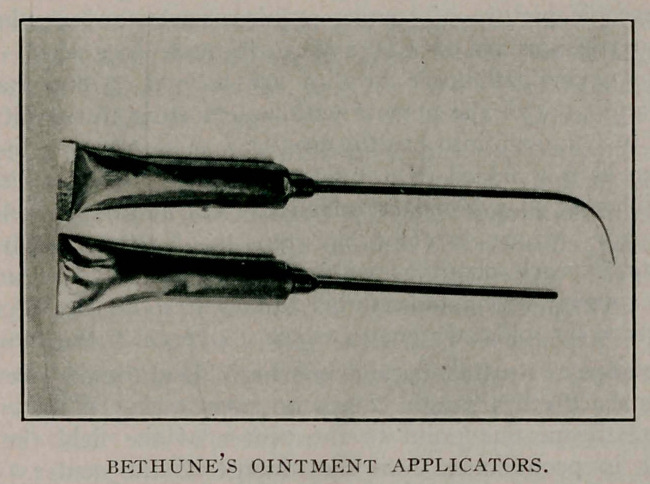# The Treatment of Chronic Gonorrhoea

**Published:** 1911-12

**Authors:** Charles W. Bethune

**Affiliations:** Buffalo, N. Y.


### The Treatment of Chronic Gonorrhoea

By CHARLES W. BETHUNE, M.D.
Buffalo, N. Y.

THERE is no sharp dividing line between the subacute and chronic stages. By chronic gonorrhoea we understand that the discharge is slight, either a drop only in the morning or, at the most, but a small drop can be expressed during the day. Pain and bodily discomfort, apart from that due to some complication, are not in evidence during this stage. The presence of profuse discharge, even though of long duration, classifies the case in the subacute stage rather than the chronic.

Chronic gonorrhoea has long been one of the bugbears of the medical profession, Ricord, the eminent French andrologist, is quoted as having said he dreamed one night that he died and went to the lower regions and his punishment was that a long file of former patients passed each pointing a gristly finger at him and repeating, "Ricord, Ricord, you could not cure my gleet." Fortunately, at present we obtain better results and need not fear a similar fate.

The urine in chronic gonorrhoea is not apt to be cloudy but the first glass almost invariably contains shreds. It must be remembered that the discarded epithelium and mucus of the healthy urethra form shreds in the urine which differ from those of gonorrhoea, only in that they contain but few leukocytes and no gonococci. A patient is sometimes seen who can squeeze a purulent drop from the meatus at all times but his urine is free from

BETHUNE'S OINTMENT APPLICATORS.

shreds; the only explanation to offer for this is that there is no mucus in the discharge and this adhesive substance being absent, the pus cells are at once disseminated throughout the urine but are too few in number to render it cloudy. This possibility warns us to centrifugalize all suspected urine which does not contain shreds. Shreds in the first glass and none in the second does not indicate that the infection is limited to the anterior urethra because the discharge is so scanty that the first jets wash out the contents of both the anterior and posterior urethra.

Filling the anterior urethra with a dilute solution of methylene blue and retaining it for a few minutes will stain all the shreds contained there and if the patient then urinates any unstained shreds, they must necessarily come from the posterior urethra.

In most cases the posterior infection has disappeared long before the anterior infection has reached the chronic stage. The most obstinate chronic cases are those complicated by vesiculitis or prostatitis but fortunately they are in the minority. The presence of a superficial infection of the posterior mucosa does not increase the obstinacy of chronic gonorrhoea.

The average chronic stage in a patient who has been treated throughout by the irrigation method does not last longer than two or three weeks. Cases which have been improperly treated during the acute and subacute stages, especially those complicated by vesiculitis and prostatitis are the most stubborn.

The urethroscope and sound are of the utmost assistance in the treatment of chronic gonorrhoea. The urethroscope enables us to inspect the entire urethra with as much accuracy as though it were the surface of the body. Correct interpretation of the urethroscopic picture of course, requires practice, but when the commoner lesions are once seen they are never forgotten. Large areas of every pathologic urethra are normal so one may become familiar with the normal without resorting to the introduction of instruments into healthy urethras. The patient's lips may be taken as a standard of normal color, dark rose in a brunette and light rose in a blond or anaemic. The mucosa of the urethra is not uniform in color but consists of alternate dark and light stripes each about as wide as a horsehair. The urethral picture roughly resembles a fluted funnel in form, the ridges being formed by folds of mucosa which converge at the center.

The edge of the tube flattens out the folds at the periphery and if it fits the urethra snugly causes an anemic ring at the circumference. From this point to the center of the field the folds increase in prominence until they merge at the center. Their convergance forms the central figure roughly * shaped when normal. Pin point depressions are seen here and there, the crypts of Morgagni. Littre's glands are too tiny to be seen by the naked eye when normal. Space does not permit an extensive discussion of the various pathologic pictures.

Patches of the mucosa whose natural gloss is diminished but with color unaltered, denote a very superficial process. Irregularity or obliteration of the folds, several folds being replaced by a single large fold, and a roughened, desquamating or granular surface, varying in color from intense red to putty yellow, denote an infiltration of the deeper layers of the mucosa with round cells and leukocytes, folliculitis is shown by the red and angry orifices of the crypts, pus often being seen issuing from them. Littre's glands, when inflammed, may occasionally be seen as tiny reddish or yellow granules.

Stricture shows a host of varied forms, from a fiery red area constricted and free from folds, to masses of cicitricial bands almost bloodless, like the scar of a burn.

The sound demonstrates strictures more accurately than the urethroscope but the bulbous bougie has been the source of many errors as it not only demonstrates strictures but also the numerous annular muscular fasiculae which exist in every urethra. Care must be taken not to mistake the normal tone of the cut-off muscle for a stricture.

Irrigations should be repeated daily, or at least on alternate days. Mild or superficial inflammations, which are seen through the urethroscope as patches of diminished gloss, yield readily to irrigations alone.

Infiltrations of round cells and leukocytes, the so-called soft infiltrations described above, are best treated by dilatation, the passage of full sized sounds, or better, the use of Kohlmann dilators every third or fourth day. Dilatation causes a pressure atrophy and subsequent hyperemia of the urethra which hastens the absorption of the pathologic infiltrate as well as squeezing out the contents of the infected follicles. Dilatation must always be followed by an irrigation to remove the infectious material expressed as well as any bacteria introduced by the instrument.

Applications of 1 per cent. silver nitrate through the urethroscope repeated every fifth day is the best treatment for granular patches. These applications are to be made with a swab and great care must be taken not to smear it over the healthy mucosa.

Folliculitis indicates dilatations which both expresses their infectious contents and stimulates their resolution.

Filing the urethra with antiseptic astringent or stimulating ointments has become popularized through the efforts of Dr. H. H. Young, especially because of the very serviceable instillator which he has invented. (John Hopkins Hosp. Rep. Vol. XIII. P115.) None of the posterior applicators being satisfactory, the writer tried an 18F catheter the tip filed off with a nipple on the other end which fitted the nozzle of an ointment tube. This proved so satisfactory that a straight tube was tried in the anterior urethra. These applicators (made by the Sands-Levy Co.), are sterilized by keeping them immersed in a 5 per cent. solution of phenol, before using the tube and applicator are held under a stream of hot water to remove the phenol and soften the ointment. If an irrigation is given it should precede the ointment application.

Anhydrous lanoline is the only satisfactory vehicle for urethral ointments as vaseline and lard do not readily penetrate the mucosa. Formulae are legion: phenol, nitrate of silver, Argyrol, Protargol, etc., are the basis of the antiseptic. I personally prefer Crede's ointment with ichthyol as an adjuvant diluted with at least ten times its quantity of lanoline. Alum and zinc sulph. are the best astringents and salicylic acid may be used as a stimulant in cases where the mucosa is replaced by thick dry epithelium. All these substances may be used in a mixture two or three times as strong as their corresponding aqueous solutions. The applications may be repeated daily or on alternate days and continued for a long time.

At this point it might be well to mention a few facts in regard to lubricants for urethral instruments. All oily substances are to be avoided as they coat the mucosa with a waterproof substance which protects it from the subsequent irrigation. There are numerous water soluble lubricants on the market having Iceland moss or gum trag as a base. The most satisfactory is Caspar's, whose formula is as follows: dissolve 00.25 Gm. of oxycyanide of mercury in 100.00Cc. of boiling water and add 3.00Gm. of gum trag ribbons; allow the mixture to macerate for a week; then stir in 30.00Cc. of glycerine, strain through gauze and sterilize by steam.

Chronic gonorrhoea complicated by vesiculitis and prostatitis is apt to prove very intractable. Vaccine should be administered every third or fifth day, the initial dose being 5,000,000; double the dose at each injection until either a reaction or improvement occurs then repeat the last dose not oftener than every seven days.

Massage of the infected prostate and vesicles is not so apt to result in epididymitis during the chronic as in the acute and subacute stages. In spite of its comparative safety at this period epididymitis occasionally results. The technic is as follows: the patient holds his urine for several hours before treatment as it is impossible to massage the vesicles unless we have them supported by a full bladder. The patient drops his trousers and leans on his abdomen over the edge of a table. The index finger, protected by a rubber cot, is lubricated with vaseline and inserted into the rectum. Each vesicle is then stripped down by several gentle strokes. The same treatment is then applied to the prostate. Massage is often followed by the expulsion of considerable mucopurulent material from the meatus. The patient then urinates and is irrigated, the bladder being filled if possible. I believe that the stimulation of the massage is of more benefit than the simple expulsion of the infectious contents. Massage may be repeated every third or fifth day. Occasionally operative drainage of the vesicle or prostatectomy is our only resort. (Fuller).

It must be borne in mind that tuberculosis of the epididymis, cord, prostate and vesicle occasionally follows a gonorrhoeal infection of these structures. The absence of night sweats, emaciation, etc. during the early stages of genito-urinary tuberculosis together with the uncertainty of the various tuberculin reactions justifies an exploratory section of the scrotum and excision of small portion of the epididymis for microscopic examination in any case which does not show marked improvement after several months of proper and persistent treatment.

Chronic gonorrhoea often taxes our patience, and our patients, also, to the utmost but only a very small proportion, if any of purely gonorrhoeal infection, are incurable. Two or three months is the average duration in cases improperly treated during the earlier stages but at least 5 per cent. require six months treatment.

Space does not permit a prolonged discussion of the proof of cure in gonorrhoea. Failure to find the gonococcus after repeated examinations of the tripper faden, sediment of centrifugalized urine, material massaged from the prostate and vessicles together with the absence of all clinical signs of inflammation. No patient should marry in less than a year after all signs of infection have disappeared as time is a very important factor in the elimination of the gonococcus.

## Figures and Tables

**Figure f1:**